# MicroRNA-27b-3p Promotes Tumor Progression and Metastasis by Inhibiting Peroxisome Proliferator-Activated Receptor Gamma in Triple-Negative Breast Cancer

**DOI:** 10.3389/fonc.2020.01371

**Published:** 2020-08-05

**Authors:** Song-Jie Shen, Yu Song, Xin-Yu Ren, Ya-Li Xu, Yi-Dong Zhou, Zhi-Yong Liang, Qiang Sun

**Affiliations:** ^1^Department of Breast Surgery, Peking Union Medical College Hospital, Chinese Academy of Medical Sciences and Peking Union Medical College, Beijing, China; ^2^Department of Pathology, Peking Union Medical College Hospital, Chinese Academy of Medical Sciences and Peking Union Medical College, Beijing, China

**Keywords:** triple-negative breast cancer, microRNA-27b-3p, PPARG, prognosis, metastasis

## Abstract

**Introduction:** The role and underlying mechanisms of miR-27b-3p in triple-negative breast cancer (TNBC) remains unclear.

**Methods:** miR-27b-3p expression level was evaluated in 99 TNBC patients with a median follow-up time of 133 months. The biological functions of miR-27b-3p by targeting PPARG were assessed by luciferase reporter assay, CCK-8 assay, Transwell assay, wound healing assay, western blot analysis and xenograft models.

**Results:** High level of miR-27b-3p expression was found to confer poor prognosis in TNBC patients. MiR-27b-3p overexpression increased TNBC cell proliferation, migration, invasion, and metastasis. Our data suggested peroxisome proliferator-activated receptor gamma (PPARG) was a target of miR-27b-3p. The capacity of miR-27b-3p to induce TNBC progression and metastasis depended on its inhibition of the PPARG expression. Furthermore, restoring PPARG expression reversed the effect of miR-27b-3p overexpression. Mechanistically, miR-27b-3p regulated metastasis-related pathways through PPARG by promoting epithelial–mesenchymal transition. By suppressing PPARG, miR-27b-3p could also activate transcription factors Snail and NF-κB, thereby promoting metastasis.

**Conclusions:** miR-27b-3p promotes TNBC progression and metastasis by inhibiting PPARG. MiR-27b-3p may be a potential prognostic marker of TNBC, and PPARG may be a potential molecular therapeutic target of TNBC.

## Introduction

Breast cancer is the major cause of cancer-related death among women worldwide (1). Triple-negative breast cancer (TNBC) is characterized by lack of estrogen receptor (ER), progesterone receptor (PR), and human epidermal growth factor receptor 2 (HER2). Compared with other subtype of breast cancer, TNBC has a highly aggressive clinical course, with earlier age of onset, greater metastatic potential, and lack of targeted therapy (2). Due to its heterogeneity, the currently used clinicopathological markers cannot accurately classify the prognosis of TNBC, and the molecular mechanisms that drive TNBC recurrence and metastasis remains to be further elucidated (3–5).

MicroRNAs (miRNAs) are a family of small, endogenous, non-coding RNAs that negatively regulate gene expression of specific mRNA targets by binding to the 3'-untranslated region (3'-UTR) complementary sequences. Deregulated miRNAs are involved in extensive cellular processes of TNBC, exerting their function as oncogenes or tumor suppressors depending on their cellular targets (6). One of these miRNAs is microRNA-27b-3p (miR-27b-3p), which has been found to primarily function as an oncogenic miRNA by targeting multiple tumor-suppressor genes in breast cancer (7). Previous studies have reported that miR-27b-3p can suppress multiple target genes, such as *NISCH* (Nischarin protein) (8), *ST-14* (suppression of tumorigenicity 14) (9), *HIC1* (hypermethylated in cancer 1) (10), *PSAP* (prosaposin) (11), and *PDHX* (pyruvate dehydrogenase protein X) (12), to promote tumor progression and metastasis in breast cancer. However, several papers reported conflicting roles of miR-27b-3p in breast cancer. In luminal-subtype breast cancer (ER-positive), miR-27b-3p was reported to be negatively regulated by ER to inhibit proliferation of breast cancer cells and high miR-27b-3p level indicated good survival (13, 14). Thus, the role of miR-27b-3p in breast cancer appears to be dependent on the molecular subtype of breast cancer. In TNBC, the role of miR-27b-3p and its target pathways in tumor progression and metastasis is not fully understood and needs to be clarified.

In the present study, we found that high expression of miR-27b-3p was associated with a high risk of early recurrence and poor survival in TNBC patients. By down-regulating its target PPARG, miR-27b-3p can promote tumor proliferation, migration, invasion, and metastasis in patients with TNBC. In addition, miR-27b-3p targets PPARG to promote TNBC progression and metastasis by regulating the epithelial–mesenchymal transition (EMT) process and activating the transcription factors Snail and NF-κB.

## Materials and Methods

### Ethical Statement

This study was approved by Peking Union Medical College Hospital Institutional Review Board. All procedures performed in studies involving human participants were in accordance with the ethical standards of the institutional and national research committee and with the tenets of the Declaration of Helsinki and its later amendments. Informed consent was obtained from all individual participants included in the study. All procedures involving animals were monitored in accordance with the ethical standards and the Care and Use of Laboratory Animal guidelines issued by the administrative government, under the protocol approved by the Institutional Animal Care and Use Committee of Peking Union Medical College. All procedures were performed in such a manner that animals did not suffer unnecessarily at any stage of experiments.

### Clinical Profile and Tissue Sample

We evaluated 99 patients who were treated for TNBC at the Department of Breast Surgery, Peking Union Medical College Hospital between September 2002 and March 2012. The inclusion criteria were: (1) a pathological type of invasive ductal breast carcinoma; (2) follow-up of at least 10 years for disease-free patients; and (3) with adequate samples in the tissue bank for further analysis. Patients with local advanced or metastatic breast cancer at diagnosis, bilateral or inflammatory breast cancer, neoadjuvant treatment before surgery were excluded. The histological diagnosis and confirmation of estrogen receptor, progesterone receptor, and HER-2 status were independently confirmed by two pathologists following the American Society of Clinical Oncology/College of American Pathologists guidelines (15). The antibodies used for immunohistochemistry were estrogen receptor (M7047, clone 1D5), progesterone receptor (M3569, clone 636), and HER-2 (A0485, polyclonal rabbit antibody) (Dako, Denmark). If a status of 2+ positive was obtained via immunohistochemistry, fluorescence *in situ* hybridization analysis using a dual-color probe (PathVysion, Vysis Inc., USA) was required to confirm the HER-2 status. All patients were administered anthracycline- and/or taxane-based adjuvant chemotherapy. Patients with more than three positive axillary lymph nodes or those who received breast-conserving surgery underwent radiation therapy. Endocrine or trastuzumab treatment was used as necessary. Radiographic imaging was conducted immediately after the diagnosis of breast cancer and on follow-up at a frequency of once every 6 months during the first 2 years after surgery and once every 12 months thereafter.

### RNA Extraction

Formalin-fixed paraffin-embedded primary tumor blocks were made immediately after the tissues were resected and fixed in 10% buffered formalin. Each of the 99 tumor blocks was stained with hematoxylin and eosin to identify the invasive ductal breast carcinoma regions. Five to ten pieces of 10-μm-thick tissue core were obtained from the invasive ductal breast carcinoma region of each formalin-fixed paraffin-imbedded block to minimize contamination from adjacent tissues. Total RNA was isolated from tumor tissue cores using the RecoverAll Total Nucleic Acid Isolation Kit (Ambion, USA) according to the protocol for miRNA expression profiling.

### Real-Time Reverse Transcription Polymerase Chain Reaction (RT-PCR)

For RT-PCR analysis of miR-27b-3p, the specific stem–looped RT-PCR primers and amplification of miR-27b-3p were designed according to a previous study (16). Real-time PCR analysis was performed on an ABI 7500 instrument (ABI Inc., USA) with 20-μL reaction volumes containing 1 μL reverse transcription product, 10 μL 2X SYBR Green Mix (Invitrogen), 0.8 μL paired specific primers (10 μM), and 8.2 μL H_2_O. The reactions were incubated in 96-well plates at 95°C for 5 min, followed by 40 cycles of amplification (95°C for 15 s, 60°C for 31 s). They were then ramped from 6 to 95°C to obtain the melting curve (17). U6 small nuclear RNA (snRNA) was measured using the same method and used for normalization. For RT-PCR analysis of PPARG, ACTB was used as a loading control. The following primers were used: miR-27b-3p: forward, 5′-GGCGTGTTCACAGTGGCTAAG-3′ and reverse, 5′-GTCGTATCCAGTGCAGGGTCCGAGGTATTCGCACTGGATACGACGCAGAA-3′; U6: forward, 5′-CTCGCTTCGGCAGCACA-3′ and reverse, 5′-AACGCTTCACGAATTTGCGT-3′; PPARG: forward, 5′-GATATCGACCAGCTGAATCC-3′ and reverse, 5′-TTGTCTGTTGTCTTTCCTGT-3′; ACTB: forward, 5′-GATGAGATTGGCATGGCTTT-3′ and reverse, 5′-GTCACCTTCACCGTTCCAGT-3′. The relative expression was calculated using the equation relative quantity=2^−ΔCT^ (18), where ΔCT is the difference in threshold cycles to detect fluorescence.

### Cell Culture and Transfection

Human TNBC cell line MDA-MB-231 was obtained from the Cell Bank of the Chinese Academy of Sciences (Shanghai, China) and cultured in Dulbecco's modified Eagle medium (Life Technologies, Carlsbad, CA) supplemented with 10% of fetal bovine serum (Life Technologies) at 37°C with 5% CO_2_. The miR-27b-3p mimics, miR-27b-3p inhibitors, and corresponding control (miRNA negative control, NC) were purchased from Shanghai GenePharma (Shanghai, China). MDA-MB-231 cells were seeded overnight and transfected with miR-27b-3p mimics, miR-27b-3p inhibitors, and miR-27b-3p NC with corresponding control the next day using Lipofectamine 2000 (Invitrogen, Grand Island, NY, USA) according to the manufacturer's instructions. After 48 h of incubation, the mRNA and protein were subsequently collected for RT-PCR and western blot analysis.

### Cell Proliferation Assay

The colorimetry method with Cell Proliferation and Cytotoxicity Assay Kit (CCK-8 assay kit, Dojindo, Japan) was used to measure cell proliferation. MDA-MB-231 cells were seeded into 96-well plates and cultured overnight. Each plate was occupied by the following groups: blank group, miR-27b-3p NC group, miR-27b-3p mimics group, miR-27b-3p inhibitors group, or miR-27b-3p mimics plus PPARG agonist group (rosiglitazone, 80 μmol/L). After 0, 24, 48, and 72 h of transfection, cells were treated with 100 μL of CCK-8 solution for 1 h of incubation at 37°C with 5% CO_2_. The optical density of each well was measured using a microplate spectrophotometer (Pulang Technology Co. Ltd, Beijing, China) at 450 nm (optical density, 450 nm).

### Transwell Invasion and Migration Assay

The invasion and migration capabilities of the cells were measured via the Transwell assay using a 24-well Transwell chamber (Corning Life Sciences, MA, USA) according to the manufacturer's instructions. For invasion assay, Transwell inserts were pre-coated with 80 μL of concretionary Matrigel (Corning Life Sciences, MA, USA). A load of 4 × 10^5^ cells were re-suspended in serum-free medium and plated in each of the upper chambers, with the bottom chambers filled with growth medium containing 0.7 mL 10% FBS. After 24 h of humidified incubation at 37°C with 5% CO_2_, the invaded cells at the bottom surface were fixed with 4% paraformaldehyde and stained with HE. Cell count was obtained using a microscope at 200 × magnification.

### Wound Healing Assay

MDA-MB-231 cells were seeded in 6-well plates with 4 × 10^5^ cells per well and grown to over 90% confluence as monolayers. The cells were scratched with a sterile tip perpendicular to the previously painted line. The wounds of scratch were photographed and measured at indicated time points of 0 and 24 h using BX41 light microscope.

### Target Gene Prediction for miR-27b-3p

Possible miR-27b-3p binding sites were obtained by a bioinformatics analysis and online miRNA target gene prediction platforms, including miRanda (https://omictools.com/miranda-tool), Targetscan (http://www.targetscan.org/), and miRDB (http://mirdb.org/). The predicted 3'-untranslated-region (UTR) sequence of PPARG was suggested as the highly conservative match for miRNA-27b-3p as target site.

### Luciferase Reporter Assay

For the luciferase reporter assay, firefly luciferase reporter plasmids containing the wild-type 3'-UTR of PPARG (Promega, WI, USA) or mutated-3'-UTR (mut-3'-UTR) of PPARG (Promega, WI, USA) were co-transfected with miR-27b-3p or NC using Lipofectamine 2000 (Invitrogen, CA, USA) into MDA-MB-231 cells. The ratio of firefly to Renilla luciferase activity was measured using the Dual Luciferase Reporter Assay System (Promega, WI, USA) 48 h after transfection, and luciferase activity was determined by normalizing firefly against NC luciferase activity.

### Protein Extraction and Western Blot Analysis

Total protein was extracted with a cell lysis reagent and the concentration was measured using a BCA protein assay kit (Beyotime Biotec, China). Protein samples were separated by 10% sodium dodecyl sulfate polyacrylamide gel electrophoresis. The separated proteins were transferred onto a polyvinylidene difluoride membrane (Millipore, MA, USA), which was then blocked with 5% skim milk powder dissolved in Tris-buffered saline-Tween. After incubation at room temperature for 1 h, the membrane was incubated overnight with primary antibodies at 4°C. The primary antibodies of anti-PPARG, anti-E-cadherin, anti-Vimentin, and anti-GAPDH were purchased from Abcam (MA, USA); anti-Snail from Gene Tex (CA, USA) and NF-κB from Cell Signaling Technology (MA, USA). The membranes were then incubated for 1 h with horseradish peroxidase-conjugated secondary antibody (Abcam, MA, USA) at room temperature. The proteins were detected using an ECL detection Kit (Millipore, MA, USA). The signal density of protein bands was quantified using the Tanon-5200 Imaging System (Tanon Science & Technology, China). GAPDH was used as a loading control.

### *In vivo* Experiments

For nude mouse xenograft model, 5-week-old female BALB/c nude mice were purchased from Cavens (Jiangsu, China) and housed for 1 week before injection. The MDA-MB-231 cells were transfected with miR-27b-3p transfection control, miR-27b-3p mimics, or miR-27b-3p mimics and treated with PPARG agonist (rosiglitazone, 80 μmol/L) for 24 h. Then, these MDA-MB-231 cells (1 × 10^7^) were mixed with 150 μl of Matrigel and inoculated subcutaneously into the mammary fat pad of female nude BALB/c mice (three in each group). The tumor models were physically examined every 4 days since the 8th day. The dimensions of each tumor were obtained, and the tumor volume was determined using the following formula: tumor volume (mm^3^) = (π × length × width^2^)/6. On the 24th day, the animals were euthanized, and the tumors were excised and weighed.

### Statistical Analyses

Disease-free survival was defined as the time from primary treatment to first recurrence (local, regional, distant, or contralateral) or death by any cause. Overall survival was defined as the time from primary treatment to death by any cause. Survival curves were drawn using the Kaplan-Meier method and compared via log-rank test. Between-group differences in categorical and continuous variables were analyzed using the chi-square test (or Fisher's exact test) and using non-parametric tests, respectively. A two-way independent Student's *t*-test was performed to analyze the results of luciferase and proliferation assays. For comparison between more than two experimental groups, the one-way analysis of variance (ANOVA) test was used. All statistical analyses were performed using SPSS software 22.0 (SPSS Inc., IL, USA) or Graphpad Prism (version 8, Graphpad Software, Inc., CA, USA). Statistical significance was assessed using two-tailed tests with an α level of 0.05.

## Results

### High Level of miR-27b-3p Expression Confers Unfavorable Prognosis in TNBC Patients

Overall, 99 TNBC patients were enrolled with a median follow-up time of 133 months (range, 120–194 months). Of them, 52 patients did not develop recurrence or metastasis, while 47 patients developed recurrent events within a median interval of 22 months (range, 2–70 months) (Table 1). There was no significant correlation between recurrence and clinicopathological factors (e.g., patient's age, tumor size, or lymphatic vessel invasions) in both groups (all *P* > 0.05), except for lymph node status (*P* = 0.001).

**Table 1:** Clinicopathological characteristics between the patients with or without recurrence.

	**Patients without**	**Patients with**	***P*-value**
	**recurrence *n* (%)**	**recurrence *n* (%)**	
Age at diagnosis (y)			0.538
≤ 50	26 (52.00%)	24 (48.00%)	
>50	26 (53.06%)	23 (46.93%)	
Tumor size (cm)			0.548
≤ 2	27 (56.25%)	21 (43.75%)	
>2	25 (49.01%)	26 (50.98%)	
Histological grade			0.323
G1	6 (60.00%)	4 (40.00%)	
G2	17 (62.96%)	10 (37.03%)	
G3	22 (45.83%)	26 (54.16%)	
Undefined	7 (50.00%)	7 (50.00%)	
LVI			0.705
No	49 (53.26%)	43 (46.73%)	
Yes	3 (42.85%)	4 (57.14%)	
P53			1.000
Negative	25 (51.02%)	24 (48.97%)	
Positive	22 (52.38%)	20 (47.61%)	
Undefined	5 (62.50%)	3 (37.50%)	
Ki-67 index^*^			0.775
<14%	9 (60.00%)	6 (40.00%)	
≥14%	34 (53.12%)	30 (46.87%)	
Undefined	9 (45.00%)	11 (55.00%)	
Lymph node Status			0.001
Negative	33 (70.21%)	14 (29.78%)	
Positive	19 (36.53%)	33 (63.46%)	

LVI, lymphovascular invasion;

* *Ki-67 index threshold of 14% was chosen according to the St. Gallen Consensus 2013*.

**Figure 1:**
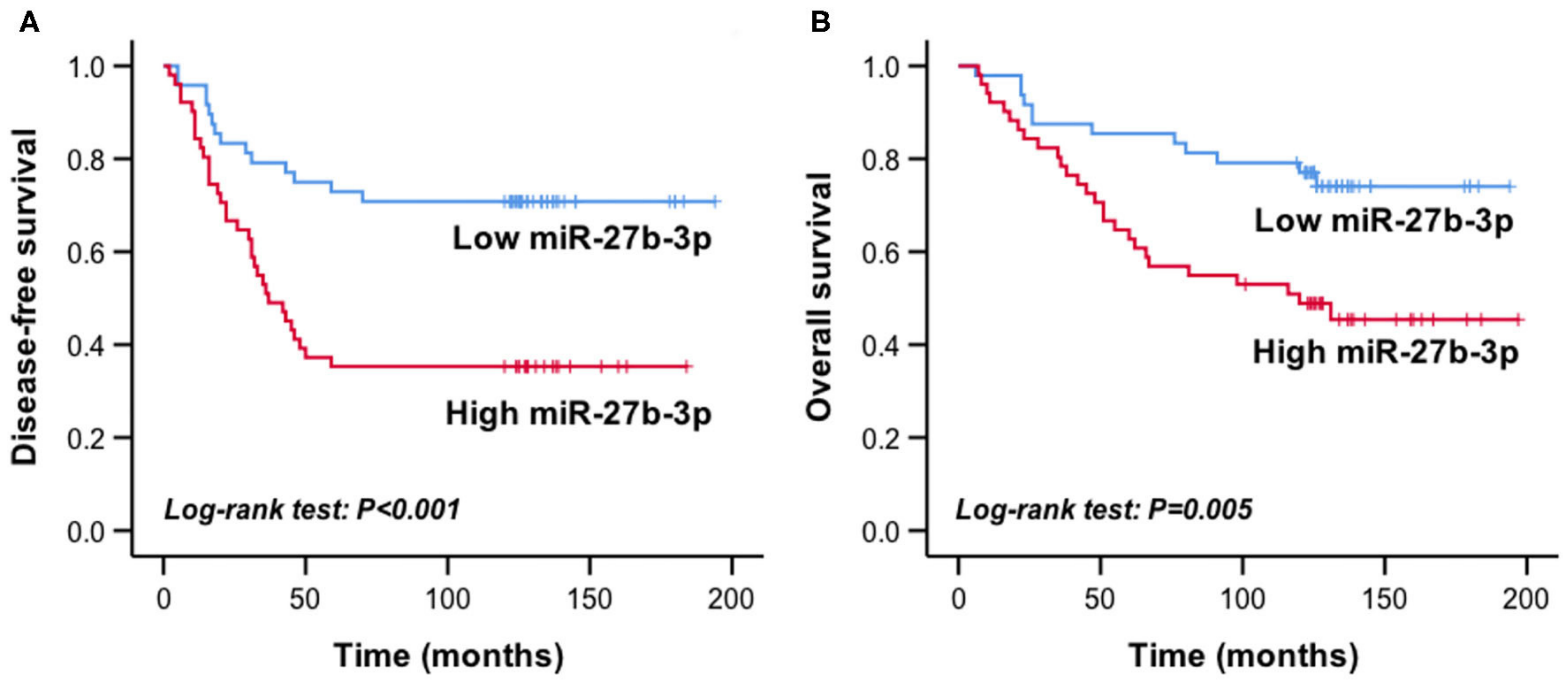
High miR-27b-3p expression indicated worse prognosis in TNBC patients. Kaplan-Meier curves showed the disease-free survival **(A)** and overall survival **(B)** of TNBC patients with high or low expression of miR-27b-3p. *P*-values were computed by a log-rank test.

The mean relative expression level of miR-27b-3p of the recurrent group was 0.224 ± 0.172, which was significantly higher than that of the disease-free group (0.136 ± 0.120, *P* = 0.004). According to the median expression of miR-27b-3p (0.127, range, 0.021–0.911), the patients were divided into the high expression group (*n* = 49) and the low expression group (*n* = 50). In Kaplan-Meier survival analysis, the high miR-27b-3p expression group had a significantly shorter disease-free survival and overall survival than the low expression group (*P* < 0.001 and *P* = 0.005, respectively, Figures 1A,B). These results suggested the potential of miR-27b-3p as a prognostic marker of TNBC.

### MiR-27b-3p Promotes TNBC Cell Proliferation, Migration, and Invasion

**Figure 2:**
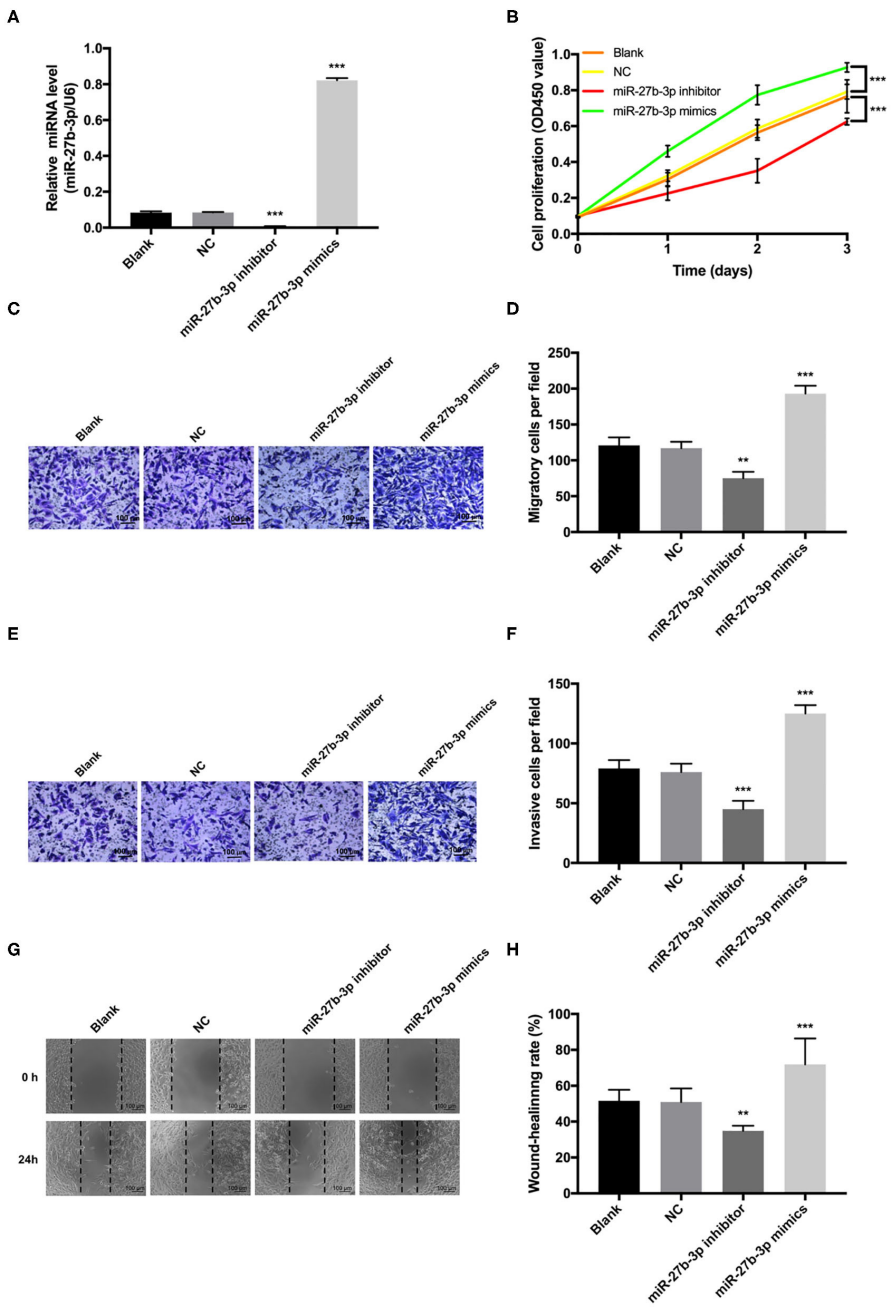
MiR-27b-3p promotes cell proliferation, migration, and invasion in TNBC. MDA-MB-231 cells were transiently transfected with miR-27b-3p mimics or inhibitors. **(A)** The relative miR-27b-3p levels were detected by RT-PCR in MDA-MB-231 cells transfected with blank, negative control (NC), miR-27b-3p inhibitors or miR-27b-3p mimics. **(B)** CCK-8 proliferation test showed that overexpression of miR-27b-3p promoted MDA-MB-231 cells proliferation and miR-27b-3p inhibitors decreased cell proliferation. **(C,D)** Transwell migration assay. Overexpression of miR-27b-3p increased cell migration significantly, while inhibition of miR-27b-3p decreased cell migration. **(E,F)** Transwell invasion assay. Similar to the cell migration experiments, a significantly higher invasion capability was detected in the miR-27b-3p mimics group. MDA-MB-231 cells transfected with miR-27b-3p inhibitors showed significant inhibition of cell invasion. **(G,H)** Wound healing assay. The wound healing rates were compared among MDA-MB-231 cells transfected with blank, NC, miR-27b-3p inhibitors, and miR-27b-3p mimics at 24 h. For **(A,B,D,F,H)**, results are shown as mean ± SD of 3 times of experiments in each group. **P < 0.01, ***P < 0.001; NC, negative control of transfection vectors.

Transfection of miR-27b-3p mimics significantly elevated the expression level of miR-27b-3p in MDA-MB-231 cells, and miR-27b-3p inhibitors decreased the miR-27b-3p expression (Figure 2A). Overexpression of miR-27b-3p significantly increased the proliferative rate of MDA-MB-231 cells, whereas down-regulation of miR-27b-3p significantly prohibited cell proliferation via CCK-8 assay (Figure 2B). Statistical analyses of the Transwell assay confirmed that miR-27b-3p enhanced both cell invasion and migration, while miR-27b-3p inhibitors lead to opposite results (*P* < 0.01, Figures 2C–F). Similar effect on cell migration was detected in the wound healing assay with the wound gap healing faster in the miR-27b-3p mimics group and the miR-27b-3p inhibitors showing a cancelation effect on the healing process (Figures 2G,H). These results proved that miR-27b-3p enhanced TNBC cell proliferation, migration, and invasion.

### PPARG Is the Potential Target of miR-27b-3p

**Figure 3:**
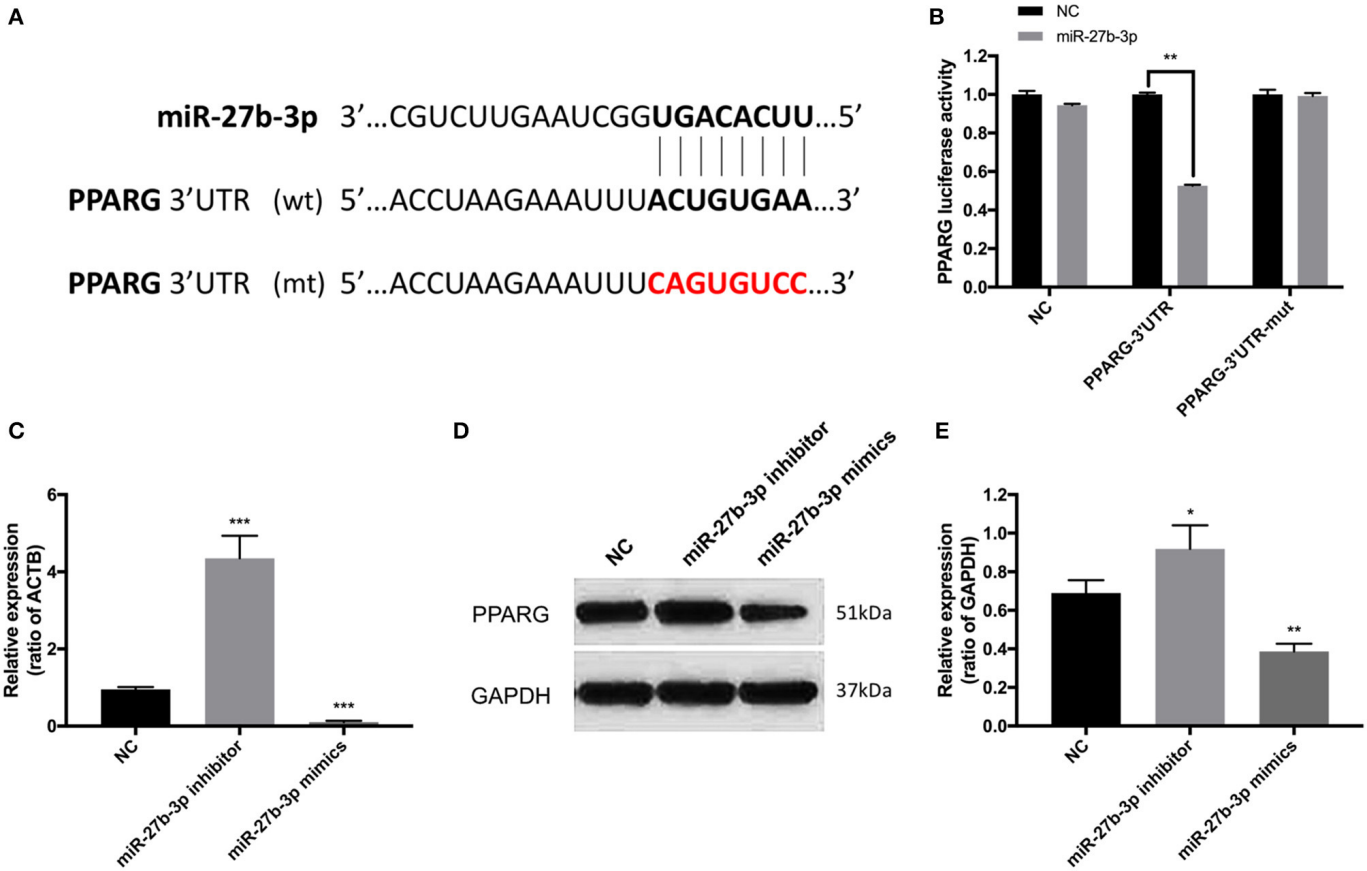
PPARG is the target of miR-27b-3p in TNBC cells. **(A)** The binding sequence of miR-27b-3p and 3'-UTR of PPARG. The mutant sequence of 3'-UTR of PPARG was generated in the complementary site for the seed region of miR-27b-3p (WT, wild type; MUT, mutant type). **(B)** Luciferase reporter assay confirmed that miR-27b-3p repressed the luciferase activity of wild-type PPARG-3'UTR, while the repression effect was not detected with mutant PPARG-3'UTR. **(C)** RT-PCR analysis showed the mRNA level of PPARG could be up-regulated by miR-27b-3p inhibitors and down-regulated by miR-27b-3p mimics (ACTB used as a loading control). **(D,E)** Western blot analysis showed that miR-27b-3p mimics inhibited the protein expression level of PPARG, while miR-27b-3p inhibitors increased the expression of PPARG (GAPDH served as a loading control). For **(B,C,E)**, results are shown as mean ± SD of 3 times of experiments in each group. *P < 0.05, **P < 0.01, ***P < 0.001; NC, negative control of transfection vectors.

A highly conservative match for miR-27b-3p with PPARG was confirmed using Internet miRNA target gene prediction platforms (Figure 3A). The 3'-UTR luciferase reporter assays further confirmed that miR-27b-3p repressed the luciferase activity of wild-type PPARG-3'UTR (Figure 3B). Compared with the transfection negative control group, the repression effect was not detected with mutant PPARG-3'UTR (Figure 3B). Furthermore, in terms of both mRNA and protein levels, PPARG expression was increased by miR-27b-3p inhibitors and reduced by miR-27b-3p mimics, confirming that PPARG expression is regulated by miR-27b-3p (Figures 3C–E).

### MiR-27b-3p Promoted Cell Proliferation, Migration, and Invasion by Inhibiting PPARG Expression in TNBC Cells

**Figure 4:**
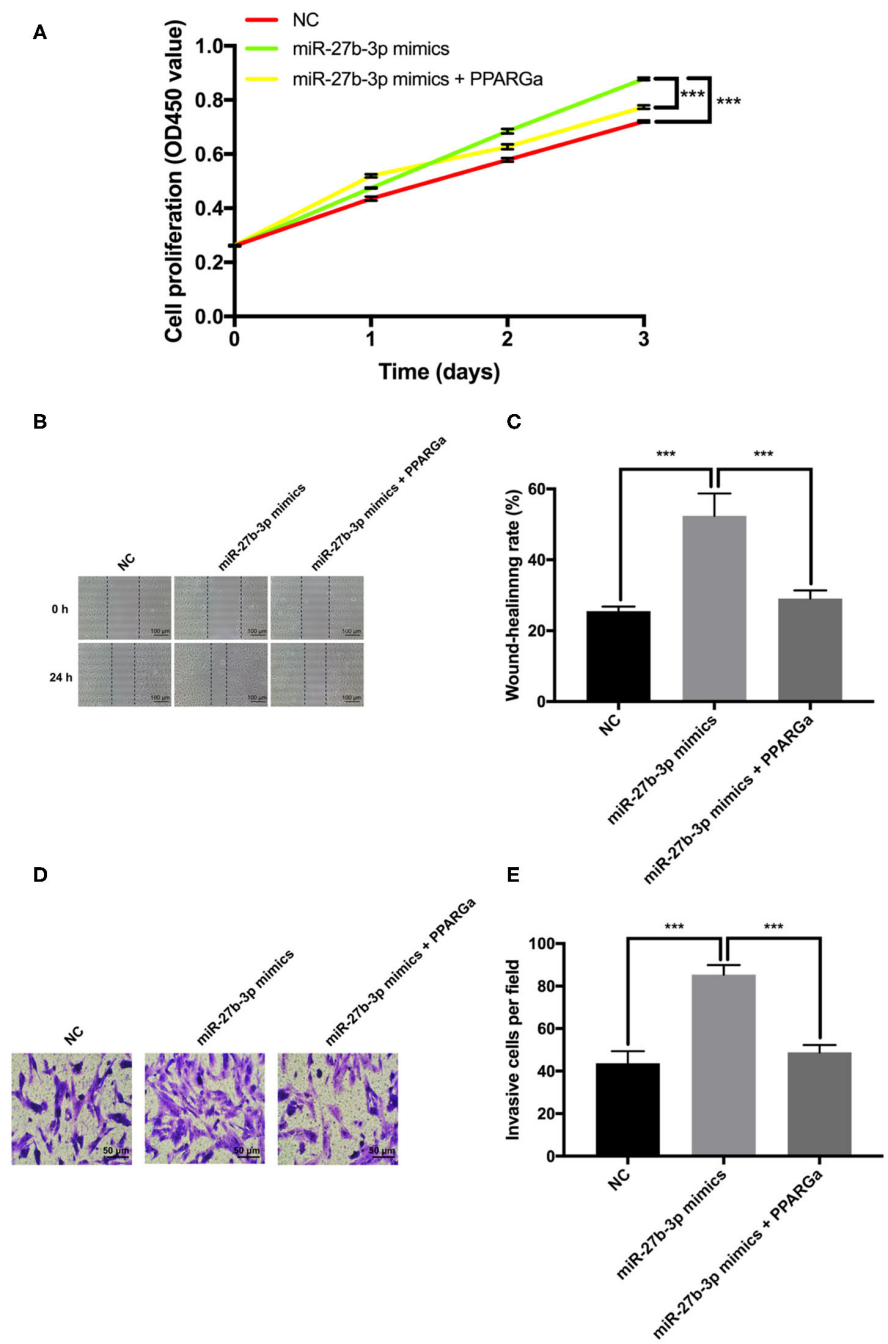
Effect of miR-27b-3p on cell proliferation and invasion depends on its inhibitory effect on PPARG expression in TNBC cells. The MDA-MB-231 cells were transfected with miR-27b-3p-3p NC, miR-27b-3p-3p mimics, or miR-27b-3p-3p mimics and treated with PPARG agonist (rosiglitazone, 80 μmol/L) for 24 h. **(A)** CCK-8 test assay showed that overexpression of miR-27b-3p could promote cell proliferation. However, this promotion effect of miR-27b-3p could be reversed by PPARG agonist. **(B,C)** Wound healing assay showed that PPARG agonist abolished the effect of miR-27b-3p mimics on the wound-healing rate of MDA-MB-231 cells. **(D,E)** Transwell invasion assay. The number of invasive cells in the miR-27b-3p mimics group were significantly higher than those of the control group, which were significantly decreased by PPARG agonist. For **(A,C,E)**, results are shown as mean ± SD of 3 times of experiments in each group. ***P < 0.001; NC, negative control of transfection vectors.

To further validate that PPARG is the functional target for miR-27b-3p while exerting its promoting effects on tumor progression and metastasis, a PPARG agonist rosiglitazone was used to restore the expression of PPARG (19, 20). CCK-8 proliferation assay demonstrated that restoration of PPARG expression significantly reversed the promotion of cell proliferation by miR-27b-3p mimics (Figure 4A). The wound healing assay showed that the wound gap in the miR-27b-3p mimics group healed faster than in the negative control group, but this seemed to be canceled by PPARG agonist, which upregulated the PPARG expression level (Figures 4B,C). Transwell invasion assay confirmed the enhancing effect of miR-27b-3p mimics on tumor invasion, while the PPARG agonist reduced this effect (Figures 4D,E). Thus, the enhancement of TNBC cell proliferation, migration, and invasion by miR-27b-3p depended on its inhibitory effect on the target PPARG.

### MiR-27b-3p Promotes Tumor Growth by Inhibiting PPARG Expression in Immunodeficient Murine Models

**Figure 5:**
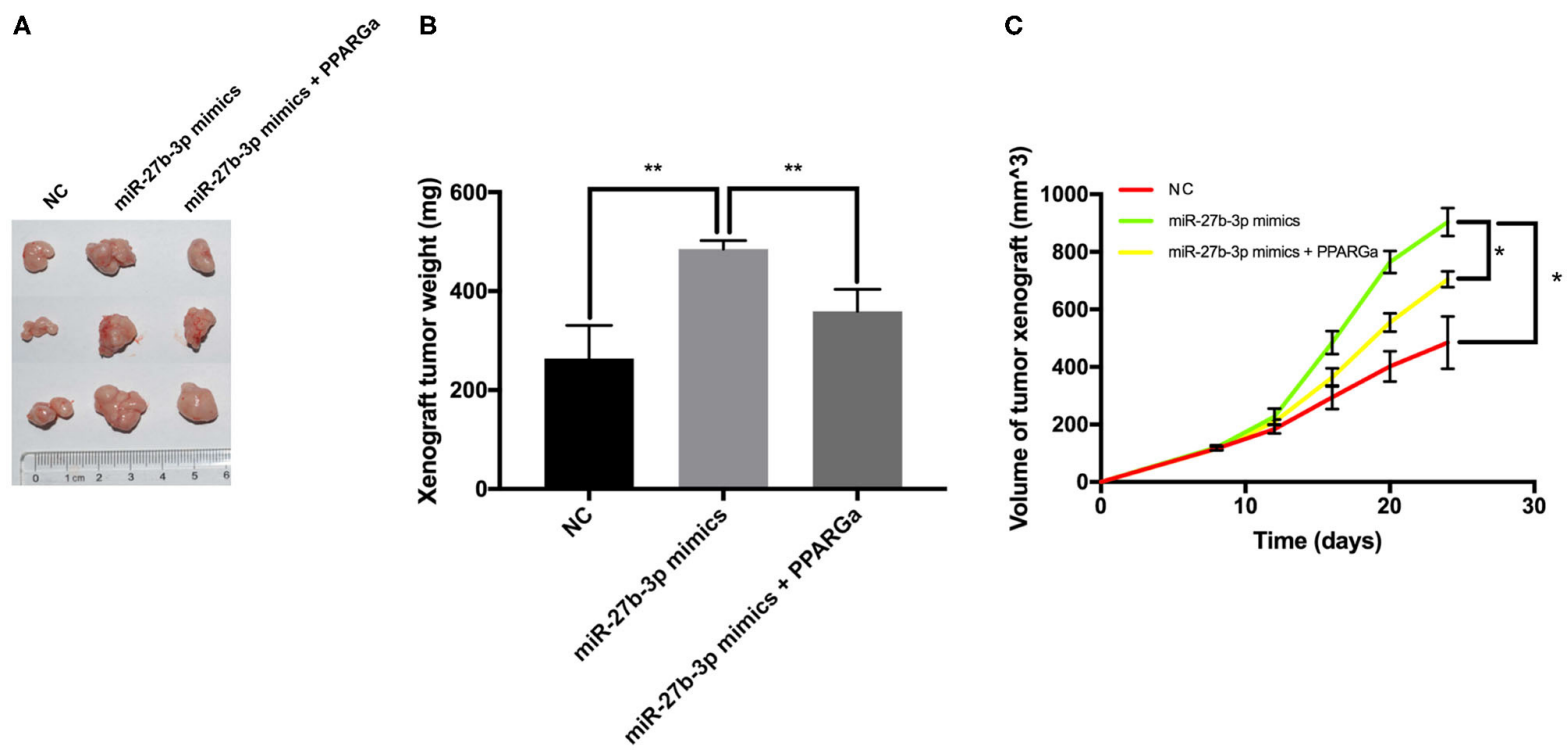
MiR-27b-3p promotes tumor growth in immunodeficient murine models. The MDA-MB-231 cells were transfected with miR-27b-3p NC, miR-27b-3p mimics, or miR-27b-3p mimics and treated with PPARG agonist (rosiglitazone, 80 μmol/L) for 24 h. Then, these cells were injected subcutaneously into the mammary fat pad of female nude BALB/c mice (three mice in each group). **(A,B)** Tumor xenografts in the miR-27b-3p mimics group significantly overweighed those in the negative control group, but the advantage was canceled by PPARG agonist. **(C)** Primary mammary tumor volume in mice injected with miR-27b-3p NC, miR-27b-3p mimics, or miR-27b-3p mimics with PPARG agonist MDA-MB-231 cells. For **(B,C)**, results are shown as mean ± SD of 3 times of experiments in each group. *P < 0.05, **P < 0.01; NC, negative control of transfection vectors.

Tumor xenografts in the miR-27b-3p mimics group overgrew and overweighed the NC group (Figures 5A,B). Compared with the NC group, the miR-27b-3p overexpression group showed a higher tumor growth rate, with a significantly larger tumor volume on the final examination (Figure 5C). PPARG agonists significantly reduced the tumor promoting effect of miR-27b-3p mimics, resulting in reduced weight and volume of tumor xenograft (Figures 5B,C). Collectively, increasing PPARG expression suppresses the miR-27b-3p oncogenic function, which plays an important role in tumor growth.

### MiR-27b-3p Targets PPARG to Regulate the EMT Process and Metastasis-Related Pathways

**Figure 6:**
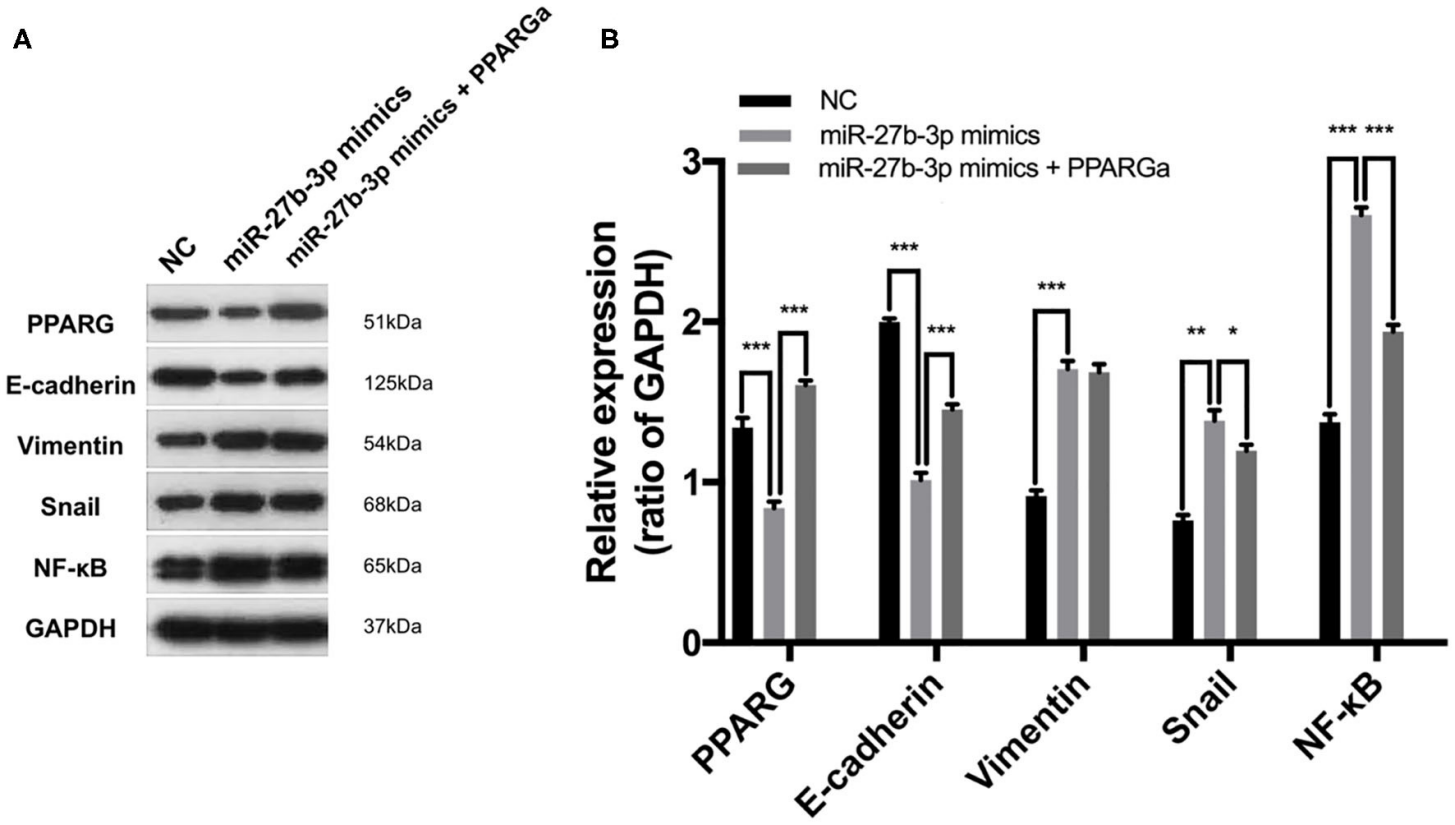
MiR-27b-3p targets PPARG to regulate the EMT process and metastasis-related pathways. **(A)** Western blot analysis of PPARG, E-cadherin, Vimentin, Snail, and NF-κB after MDA-MB-231 cells were transfected by miR-27b-3p mimics or miR-27b-3p mimics together with PPARG agonist (GAPDH served as a loading control). **(B)** Relative expression analysis showed miR-27b-3p mimics activated EMT process and activated the expression of the metastasis-related transcription factors, while these effects were significantly repressed by PPARG agonists. For **(B)**, results are shown as mean ± SD of 3 times of experiments in each group. *P < 0.05, **P < 0.01, ***P < 0.001; NC, negative control of transfection vectors.

The EMT process, which is characterized by the loss of E-cadherin and gain of Vimentin expression, is frequently activated during cancer invasion and metastasis in TNBC (21). By inhibiting the expression of PPARG, miR-27b-3p mimics significantly activated the EMT process, which was proven by down-regulated E-cadherin and up-regulated Vimentin expression in TNBC cells (Figures 6A,B). However, the activating effect of miR-27b-3p on the EMT was significantly reduced by the restoration of PPARG (Figures 6A,B). MiR-27b-3p mimics elevated the expression of the transcription factors Snail and NF-κB, while PPARG agonists significantly repressed these effects. Snail and NF-κB pathways can activate the expression of invasion-associated genes and are involved in the EMT (21), thereby promoting metastasis. These results indicate that the miR-27b-3p targets PPARG to play a critical role in TNBC metastasis by regulating the EMT process and metastasis-related pathways.

## Discussion

TNBC is an aggressive breast cancer subtype that is characterized by early metastasis and unfavorable prognosis. In our TNBC cohort, 47 of 99 patients developed breast cancer recurrence. Further, only one of those occurred on the 70th month, while all other events were detected within 5 years after the surgery. In fact, 73.1% of the events occurred within 3 years of diagnosis. Compared with other breast cancer subtypes, this phenomenon of differing pattern in TNBC metastasis implied that it may be driven by certain factors through a distinct mechanism different from the common ones.

In the present study, TNBC patients with high miR-27b-3p expression levels were found to have early recurrence and poor survival, which was consistent with other reports (22, 23). We also found that miR-27b-3p can promote tumor cell proliferation, progression, and metastasis in TNBC, which was in agreement with the oncogenic function of miR-27b-3p (8–12). Since miRNAs are able to target numerous different transcripts, it is not surprising that miR-27b-3p was reported to exert seemingly conflicting roles in different tumor cells. MiR-27b-3p was reported to inhibit proliferation and metastasis in ER-positive breast cancer (13, 14). The tumor suppressive function of miR-27b-3p in ER-positive breast cancer may result from hypermethylation in the miR-27b-3p promoter region, affecting the targets and downstream signaling pathways of miR-27b-3p (13, 24). MiR-27b-3p might work through various mechanisms by targeting different genes in a cell-type dependent manner, and thus the potential targets and pathways of miR-27b-3p in promoting TNBC progression and metastasis were investigated.

Possible miR-27b-3p targets were searched by a bioinformatics analysis and online miRNA target gene prediction platforms, including miRanda, Targetscan, and miRDB. PPARG was identified as the potential target of miR-27b-3p and confirmed by the luciferase assay. PPARG is a nuclear receptor that functions as a transcription factor to regulate the expression of several genes involved in lipid metabolism, glucose homeostasis, and tumor progression in many tissues (25–27). Our *in vivo* and *in vitro* studies provided evidence that PPARG was a functional target of miR-27b-3p in TNBC. The present study and other research (28–32) showed that PPARG acted as a tumor suppressor and was demonstrated to suppress cell proliferation, migration, and invasion in breast cancer. An evaluation of 3 951 breast cancer patients showed that patients with high levels of PPARG expression had a significantly higher survival rate than those with low PPARG expression (28). In addition, applications of PPARG agonist, rosiglitazone, as anticancer treatment in clinical trials was conducted in a pilot trial (19) and rosiglitazone was shown to reduce breast cancer risk in female patients with type 2 diabetes mellitus (33). Another study also showed that PPARG, as a target of miR-27b-3p, played a significant role in suppressing cervical cancer progression (34). However, one study showed that miR-27b-3p targeted PPARG to inhibit tumor growth and progression in neuroblastoma cells (35). Thus, miR-27b-3p and PPARG can act either as oncogenes or tumor suppressors depending on the cell type.

To explore the mechanisms of the oncogenic role of the miR-27b-3p, by suppressing PPARG in TNBC, we further investigated the possible downstream pathways. TNBC is characterized by early relapse and most importantly, frequent distant metastasis, which is always correlated with aberrant activation of EMT (21, 36). In the present study, the overexpression of miR-27b-3p reduced PPARG expression and further activated the EMT processes and metastatic pathways including Snail and NF-κB activation. Restoration of PPARG expression by a PPARG agonist could diminish the effect of miR-27b-3p overexpression on activation of the EMT processes and metastatic pathways including Snail and NF-κB. Thus, miR-27b-3p targets PPARG to promote TNBC invasion and metastasis.

In conclusion, we found miR-27b-3p might be used as a prognostic marker for TNBC patients. MiR-27b-3p was associated with migration and invasion of TNBC cells by targeting PPARG, which could activate the EMT processes and metastatic pathways. This study provides new insights into the roles of miR-27b-3p-PPARG-EMT pathways in TNBC progression and metastasis, which may be helpful for the development of future treatments for TNBC.

## Data Availability Statement

The datasets generated for this study are available on request to the corresponding author.

## Ethics Statement

The studies involving human participants were reviewed and approved by Peking Union Medical College Hospital Institutional Review Board. The patients/participants provided their written informed consent to participate in this study. The animal study was reviewed and approved by Institutional Animal Care and Use Committee of Peking Union Medical College.

## Author Contributions

S-JS: conceptualization, methodology, formal analysis, writing, editing, reviewing, and funding acquisition. YS: conceptualization, methodology, investigation, data curation, and writing. X-YR: methodology, investigation, and resources. Y-LX: investigation, resources, and editing. Y-DZ: methodology and reviewing. Z-YL: methodology and reviewing. QS: conceptualization, methodology, reviewing, project administration, and supervision. All authors contributed to the article and approved the submitted version.

## Conflict of Interest

The authors declare that the research was conducted in the absence of any commercial or financial relationships that could be construed as a potential conflict of interest.
